# A physicochemical descriptor-based scoring scheme for effective and rapid filtering of kinase-like chemical space

**DOI:** 10.1186/1758-2946-4-4

**Published:** 2012-02-08

**Authors:** Narender Singh, Hongmao Sun, Sidhartha Chaudhury, Mohamed Diwan M AbdulHameed, Anders Wallqvist, Gregory Tawa

**Affiliations:** 1 DoD Biotechnology High Performance Computing Software Applications Institute, Telemedicine and Advanced Technology Research Center, U.S. Army Medical Research and Materiel Command, Fort Detrick, MD 21702, USA; 2 NIH Chemical Genomics Center, 8900 Medical Center Dr, Rockville, MD 20850, USA

## Abstract

**Background:**

The current chemical space of known small molecules is estimated to exceed 10^60 ^structures. Though the largest physical compound repositories contain only a few tens of millions of unique compounds, virtual screening of databases of this size is still difficult. In recent years, the application of physicochemical descriptor-based profiling, such as Lipinski's rule-of-five for drug-likeness and Oprea's criteria of lead-likeness, as early stage filters in drug discovery has gained widespread acceptance. In the current study, we outline a kinase-likeness scoring function based on known kinase inhibitors.

**Results:**

The method employs a collection of 22,615 known kinase inhibitors from the ChEMBL database. A kinase-likeness score is computed using statistical analysis of nine key physicochemical descriptors for these inhibitors. Based on this score, the kinase-likeness of four publicly and commercially available databases, i.e., National Cancer Institute database (NCI), the Natural Products database (NPD), the National Institute of Health's Molecular Libraries Small Molecule Repository (MLSMR), and the World Drug Index (WDI) database, is analyzed. Three of these databases, i.e., NCI, NPD, and MLSMR are frequently used in the virtual screening of kinase inhibitors, while the fourth WDI database is for comparison since it covers a wide range of known chemical space. Based on the kinase-likeness score, a kinase-focused library is also developed and tested against three different kinase targets selected from three different branches of the human kinome tree.

**Conclusions:**

Our proposed methodology is one of the first that explores how the narrow chemical space of kinase inhibitors and its relevant physicochemical information can be utilized to build kinase-focused libraries and prioritize pre-existing compound databases for screening. We have shown that focused libraries generated by filtering compounds using the kinase-likeness score have, on average, better docking scores than an equivalent number of randomly selected compounds. Beyond library design, our findings also impact the broader efforts to identify kinase inhibitors by screening pre-existing compound libraries. Currently, the NCI library is the most commonly used database for screening kinase inhibitors. Our research suggests that other libraries, such as MLSMR, are more kinase-like and should be given priority in kinase screenings.

## Background

Chemical space can be defined as "the total descriptor space covered by all the known and possible small organic compounds" [1]. Chemical space is thus so vast that it prompted Lipinsky and Hopkins to compare it to the total number of stars in the cosmos [2]. Estimates of the total number of possible small molecules vary from 10^8 ^to 10^200 ^depending upon the criteria used. For example, Bohacek et al. [3] estimated it to be 10^60^, when based on a maximum number of 30 C, N, O, and S atoms; Ertl [4] estimated a total of 10^20^-10^24 ^possible small molecules, based on current synthetic methods; and Ogata et al. [5] estimated a range of 10^8^-10^19 ^possible small molecules, based on combinations of known Protein Data Bank (PDB) ligands.

The CAS registry [6] is the largest collection of disclosed chemical substance information and currently contains more than 55 million organic and inorganic compounds. Other notable collections of compounds include the Chemical Structure Lookup Service (CSLS) [7], with around 46 million unique compounds, PubChem [8] and Chemspider [9], with around 20 million compounds each, and ZINC [10] with around 13 million compounds, along with hundreds of other public or private collections ranging from a few thousands to a few millions of compounds. Even though such vast collections only constitute a small fraction of possible chemical space, it is still very difficult to apply a typical biological screen to all molecules in a collection when seeking novel hits on targets of interest [11]. Along with database size, another concern is that very few compounds in these databases are biologically relevant; in other words, the sub-regions of chemical space that are relevant to biology is small [1,12]. Since not every region of chemical space defined by a compound database is biologically relevant, screening the entire database for a particular target is a waste of resources. In recent years, the focus has shifted away from screening large compound libraries to screening smaller, more target-focused libraries that are generated using all relevant information about the target and its known active compounds [13-17].

The design of focused libraries using physicochemical-based descriptors is known as chemography. The underlying principle of this technique is that structurally similar compounds are likely to have similar interactions with associated targets, along with having similar physicochemical property ranges [18-22]. Such profiling of compounds based on physicochemical descriptors has been in use since the late 1990's and many excellent research articles on this concept exist [23-34]. The most popular methods are the rules defining drug-likeness proposed by Lipinski et al. [35] and more recently by Veber et al. [31] and Oprea et al. [11,29,36,37]. These rules are based on simple physicochemical descriptors such as molecular weight, number of hydrogen bond donors and acceptors, logP, polar surface area, and number of rotatable bonds. Since their publication, these rules have been extensively used to differentiate between drugs, lead-like compounds, and other compounds, and have also been used as filters to reduce the size of screening databases. Ideally, these rules must be based on individual target-based known small molecule exemplars. Previously such rules have been applied in a few target classes like G protein-coupled receptors (GPCRs) and kinases [38,39]. The ultimate goal of such applications is to maximize the diversity of hits while minimizing the size of screening libraries to cover only the biologically relevant space for a particular drug target.

Our focus in this work is to apply physicochemical descriptor-based filters to make kinase-likeness rules. Previously, to make a kinase-focused library, two main types of approaches have been used: 1) scaffold-based design, [40-45] or 2) 2D and 3D (pharmacophore) similarity-based design [46-48]. Both approaches utilize information from known kinase active and inactive compounds. While these approaches have been successfully used in test cases, their utility in finding novel active compounds is limited due to fact that the new compounds selected are often structurally similar to the compounds that are initially used as templates. In comparison, our method, which is based on physicochemical descriptors of known active compounds, has the advantage of being able to select novel structural scaffolds since the physicochemical properties are not directly tied to any specific chemical scaffold. The method is also easy to implement and can handle very large compound data sets.

Protein kinases, which are important signaling enzymes that catalyze a phosphoryl transfer from ATP to a given protein or peptide substrate, are the second most popular drug targets after GPCRs and account for roughly one third of all drug discovery projects [49]. There are currently more than 518 distinct human protein kinase genes spread across seven kinase families [50]. Each kinase contains a conserved catalytic domain consisting of N-terminal and C-terminal lobes connected by a hinge region [51]. The ATP-binding site is located in a deep cleft between these lobes. Numerous structural studies of kinases have shown that, although there is substantial diversity in sequences, the structure of the ATP-binding site [52] is relatively conserved. Over the years, thousands of inhibitors (actives) have been identified for a variety of kinases. Most of these actives compete with ATP for the ATP-binding site and are classified as ATP-mimetics. The conserved nature of the binding site architecture among kinases suggests that kinase actives occupy a common, biologically relevant region of chemical space that is compatible with the structural and chemical constraints of the ATP-binding site topology. This region can be roughly described by the common steric and electronic features, i.e., physicochemical properties, among known ATP-mimetic kinase actives [39,52]. A rigorous, high-throughput, statistical approach for identifying these features presents the opportunity to design kinase-focused libraries which consist of compounds that contain complementary features to the common ATP-mimetic actives.

To this end, we collected a set of 22,615 known kinase actives from the ChEMBL database [53]. This database was assembled from more than 1,200 published research articles. We then tested a range of physicochemical descriptors and identified those that significantly distinguish between kinase and non-kinase active compounds. The measured distributions of these properties from kinase actives was then used to train a kinase-likeness scoring function. We used this scoring function to rank order the following small molecule databases for their kinase-likeness: The National Cancer Institute database (NCI), [54] the Natural Products database (NPD), [10] and the National Institute of Health's Molecular Libraries Small Molecule Repository (MLSMR), [55] all of which are commonly used in screening for novel kinase actives. We further validated our results by rank ordering the same databases using another correlation matrix-based scoring function originally developed by Ohno et al. [56].

Finally, we used the kinase-likeness scoring function to generate a kinase-focused library. In order to demonstrate the utility of this approach, we employed a docking-based screen of this focused library against three diverse and well-known kinase targets to see if the screening results are enriched for tighter binding compounds relative to standard screening libraries. We found that the top compounds from the kinase-focused library indeed made better contacts with the kinase binding pockets than the top compounds from standard screening libraries.

Results show that our kinase scoring function, based on physicochemical descriptors, successfully generates kinase-focused libraries. As a result, application of this scoring function can be a useful first step in kinase actives lead discovery workflows.

## Materials and methods

### Database Collection

Experimentally validated kinase actives were compiled from the ChEMBL compound database, hereafter referred to as the kinase binding database (KBD). A compound was defined as a kinase active only if its K_i _or IC_50 _value was less than 10 μM. To test kinase-likeness of the compounds that are frequently used in virtual screening of kinase inhibitors, the following publicly available small-molecule databases were also collected: the National Cancer Institute database (NCI), the Natural Products database (NPD), the National Institute of Health's Molecular Libraries Small Molecule Repository (MLSMR). Additionally, for comparison, compounds from the World Drug Index (WDI) database were also collected since they cover a wide range of known chemical space. Both the NCI and the NPD databases were downloaded from the ZINC UCSF collection [57] of compounds, while the MLSMR compounds were downloaded from the PubChem database, [58] and the WDI was obtained from Thomson Reuters [59]. The data preprocessing and descriptor calculations were done with Chemical Computing Group's Molecular Operating Environment (MOE), v. 2010.10, [60] and implemented through Accelrys Pipeline Pilot, v. 8.0. [61]. The preprocessing steps included removing counterions and solvent molecules, setting protonation states and removing metal-based structures. Molecules not in the weight range of 100-700 daltons were also removed from the databases. The final prepared databases numbered 22,615 (KBD), 264,554 (NCI), 103,668 (NPD), 343,605 (MLSMR), and 61,368 (WDI) compounds.

Properties previously used by Oprea et al. [29] in formulating lead-likeness were used as the starting point. These properties included molecular weight (Wt), octanol/water partition coefficient (SlogP), topological polar surface area (TPSA), and number of rotatable bonds (RB) (bonds were considered rotatable if they a) had a bond order of 1, b) were not a ring and c) had at least two heavy neighbors). Additionally, the number of rings (Rings), hydrogen bond acceptors (HBA), hydrogen bond donors (HBD), number of nitrogen atoms (nN), and number of oxygen atoms (nO) were also included.

### Kinase-likeness Scoring Functions

For each KBD physicochemical property, there is a trend towards a normal distribution (details in Results section). Therefore, we utilized a multi-parameter Gaussian scoring function, called the Kinase-Like Score (*KLS*), for "kinase-likeness". This function is given by:

(1) $$KLS=\sum_{i=1^{e}}^{9}-\left[\frac{{\left(X_{i}-\mu_{i}\right)}^{2}}{\sigma_{i}^{2}}\right]$$

The values X*_i _*, with *i *= 1 to 9, represent the physicochemical properties of Wt, RB, HBA, HBD, SlogP, TPSA, Rings, nN, and nO, respectively. The parameters *μ*_i _and *σ*_i _represent the average and standard deviation of each physicochemical property, X*_i_*. The averages and standard deviations were calculated using property distributions obtained from the KBD. In addition, for any compound that fell outside the P10-P90 range (the descriptor range that covers 80% of the compounds) in any of its descriptors, a value of zero was assigned for that descriptor's contribution to the overall score. The *KLS *score ranges from 0 to 9, and compounds with scores toward the upper end of this range are more kinase-like than compounds with scores toward the lower end of this range. This scoring scheme shares similarities to that published by Wager et al. [62]. In their work, multi-parameter scoring functions were derived for central nervous system compounds using the properties ClogP, ClogD, Wt, TPSA, HBD, and pKa. More elaborate schemes are found in papers such as that by Oashi et al. [63] that describe a multivariate Gaussian probability distribution to ascertain bioavailability and drug likeness of small molecule compounds.

We calculated the *KLS *for all of the compounds in the NCI, NPD, MLSMR, and WDI databases. High *KLS *values of compounds in these libraries indicate that these libraries are enriched with compounds having similar physicochemical properties as those of known kinase actives and ultimately may yield a higher enrichment of kinase actives in screening.

### Correlation Coefficient-based Scoring

In addition to the *KLS*, we applied another orthogonal statistical approach based on a correlation coefficient-based methodology to rank various databases for kinase-likeness. A similar approach has already been applied by Ohno et al. to rank order various test databases for drug-likeness [56]. In this methodology we first computed the correlation coefficient between every pair of descriptors in our descriptor set using Pipeline Pilot software. This was done for the NPD, NCI, MLSMR, WDI, and KBD databases. Nine descriptor values were considered, therefore a total of 36 correlation coefficient values were determined for each database. In theory, if the correlation matrices of two compound libraries are similar, compounds in each library would be similar too. Therefore, a compound library with a correlation matrix similar to the KBD should include kinase-like molecules.

In this study, a scoring function of kinase-likeness based on a comparison of correlation matrices (*KLSC*) was defined as follows [56]:

(2) $$KLSC=\sum_{i\neq j}\left(\frac{\left|C_{i,j}\left(test\;database\right)-C_{i,j}\left(kinase\;database\right)\right|}{36}\right)$$

In this equation, *C_i, j _*(test database) and *C_i, j _*(kinase database) represent the correlation coefficient values between two descriptors *i *and *j *in the test database and kinase database, respectively. Finally, we calculated a *KLSC *value for each of the databases, NPD, NCI, and MLSMR with respect to the KBD.

### Receiver Operating Characteristic (ROC) Plots

To determine the kinase-likeness of any given database (test database) of compounds, we randomly chose 1% of the known kinase compounds from the KBD and seeded them into the NCI, NPD, MLMSR, and WDI databases. The *KLS *was computed for each compound in the seeded databases. The compounds were then sorted in decreasing order by their *KLS *value, and a ROC plot was constructed based on this ordering. The step of mixing a random selection of kinase actives from the KBD was repeated 10 times, and the average, maximum, and minimum ROC curves and area under the curve (AUC) were computed. For validation, a random 1% of kinase actives were also extracted from the KBD and mixed back with the KBD database to make ROC plots. This strategy should result in a ROC curve corresponding to a random selection of compounds from the database. To determine if a different percentage of seeded kinase compounds from the KBD gives different results, we tried other percentages as well (2%, 5%, 10%, 50%, and 100%). We found that the AUC value remains more or less independent of percentage of seeded kinase actives. As a result, only a selection of 1% was used.

### Focused Library Construction, Target Selection, and Glide Docking

We applied the *KLS *(Equation 1) to make a target-focused library for kinases. For this, we initially pooled the major databases (NCI, NPD, and MLSMR) together. From this pool of 711,827 compounds, all the known kinase actives and duplicates were removed and a kinase-focused library was constructed by selecting the top 10,000 compounds based on their kinase-likeness score (*KLS*). This set is called the focused set. Another test set of 10,000 randomly selected compounds was also selected and called the random set. To test enrichment, docking-based virtual screening (VS) was performed against three kinase targets.

For docking calculations, the receptor and ligand structures were prepared using the Protein Preparation Workflow implemented in the Maestro graphical user interface of the Schrodinger program suite. The various steps involved in this process included removing water molecules, protonating receptor and ligand, assigning optimal tautomeric states of histidine residues, flipping side chains of glutamine and asparagine residues if required, optimizing hydrogen bonds, and energy minimization. The native ligands, along with compounds from the focused set and random set, were processed using LigPrep [64] with Epik [65] to expand the protonation and tautomeric states in the range of 7.0 ± 2.0 pH units. Computational docking calculations were done using GLIDE in standard precision (SP) mode [66] for flexible docking. The binding affinities were estimated as a Docking score (Glide score + Epik state penalties). The docking protocol was validated by re-docking the native ligands, each of which reproduced the binding mode observed in the respective crystal structures. Both the focused-set and random-set compounds were docked, and the top hits were selected using a docking score cutoff value equal to the docking score of the native ligand in each crystal structure. The number of kinase ligands with respect to the total number of ligands in the resulting hit list is an indication of library enrichment.

Additionally, to show that the compounds in the focused library constructed using the physicochemical property based filtering are more diverse than scaffold-based or similarity-based focused libraries, we conducted a test. We first selected the Aldrich Market Select (CNC-AMS) library available through ChemNavigator's iResearch library [67]. This library contains ~ 7 million commercial compounds, all of which are readily purchasable. To test for diversity of focused libraries, we used an example of an already approved kinase drug Sunitinib. Sunitinib is a small-molecule targeting receptor tyrosine kinase and is approved by the FDA for the treatment of both renal cell carcinoma and the imatinib-resistant gastrointestinal stromal tumor [68]. Using Sunitinib's chemical structure, we created three types of focused libraries. First, we created a scaffold-based library by collecting all the compounds from CNC-AMS that contain the same cyclic scaffold as Sunitinib. Second, we created a similarity-based library by collecting the top 100 most similar compounds to drug Sunitinib from CNC-AMS based on Tanimoto similarity score. Third, we created a physiochemical property -based focused library by applying our KLS score and selecting the top 100 KLS scoring compounds from the CNC-AMS library. To evaluate the diversity of each library, we calculated the average of distance, where distance is defined as '1 - similarity', for every pair of collected compounds in all the three libraries. Extended-connectivity fingerprints_6 (ECFP_6) were used to calculate the similarity [69]. For comparison, we also calculated the average distance of sets of 100 randomly selected molecules from the CNC-AMS database.

## Results

### Molecular Descriptors

Studies based on Lipinski's rule-of-five [35] and Oprea's lead-like criteria [11] have established that specific sets of properties, such as size, solubility, flexibility, polarity, and hydrogen bond capacities, are sufficient to capture significant molecular features and, in turn, the biological properties of small molecules. In this study, we used nine physicochemical descriptors that influence the above-described properties to effectively quantify the kinase-likeness of a given small molecule. Out of these nine descriptors, six (Wt, RB, HBA, HBD, TPSA, and SlogP) were used by Oprea [11]. In the case of kinase targets, we have prior knowledge of binding site architecture, so three additional descriptors (Rings, nN and nO) were added. These were included because planar ring scaffolds are known to fit well in the ATP binding pocket, and nitrogen and oxygen atoms play a large role in making hydrogen bond contacts.

**Figure 1:**
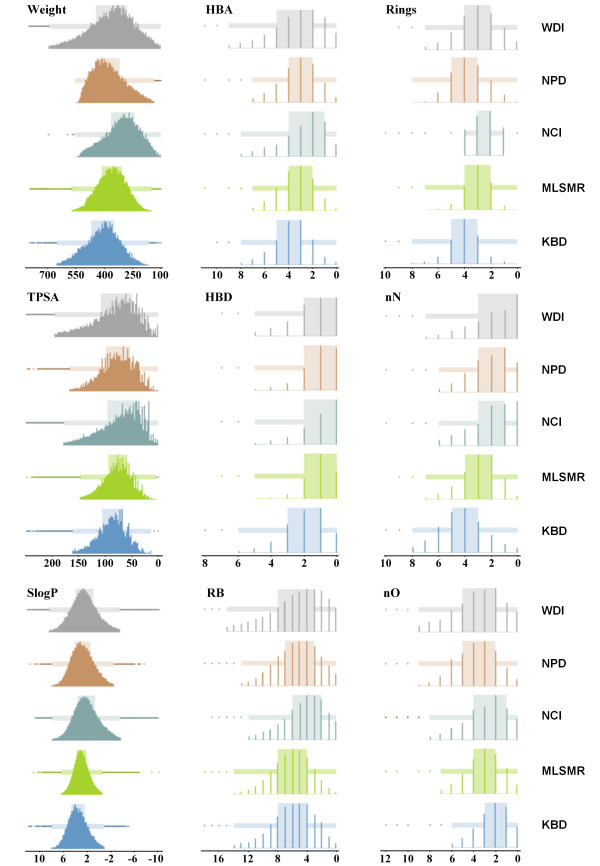
**Distribution maps of nine calculated physicochemical descriptors for five databases: WDI (grey), NPD (orange), NCI (teal), MLSMR (green), and KBD (blue)**. Each box plot shows a wide rectangular block of interquartile range (IQR) between the 1^st ^quartile and 3^rd ^quartile (covering 50% of the total range). A long, narrow, solid rectangle is also shown within the IQR, indicating upper and lower adjacent values (UAV and LAV). The dots beyond UAV and LAV are outliers.

We compared the distribution of these descriptors for kinase actives (KBD database) with those of molecules from the NPD, NCI, MLSMR, and WDI databases. Comparison was done using a two-sided Student's *t *test [70] to determine if the difference in descriptor distributions between two databases was significant. The analysis showed that, with the exception of the number of RB between the KBD and MLSMR databases (a p value of 0.528), all other descriptors were significantly different (p < 0.05). Hence, these nine different descriptors are suitable for formulating a scoring function that can differentiate between small molecules that are likely to bind kinases (actives that have kinase-likeness) and those that are not. The distribution plots of the nine descriptors, along with the outliers, average values, standard deviation (SD) is shown in Figure 1 and Table 1. A complete table with included P10-P90 (range covering 80% of the compounds) values is provided in the Additional File 1.

**Table 1:** Statistical parameters based on the distribution maps of nine calculated physicochemical descriptors for five databases (DB): WDI, NPD, NCI, MLSMR, and KBD.

DB	Total	Outliers	Avg	SD	Outliers	Avg	SD	Outliers	Avg	SD
		**Weight**			**HBA**			**Rings**		
**WDI**	61368	1145	379	138	2469	3.6	2.7	656	3.1	1.7
**NPD**	103668	198	375	86	1264	3.2	1.6	0	3.6	1.3
**NCI**	264554	38	305	91	2161	2.8	1.9	34368	2.5	1.4
**MLSMR**	343605	2544	362	78	1843	3.4	1.5	131	3.0	1.0
**KBD**	22615	250	411	96	140	3.7	1.6	32	4.0	1.2
										
		**TPSA**			**HBD**			**nN**		
**WDI**	61368	2305	89	54	2511	1.7	1.8	747	1.9	1.9
**NPD**	103668	1299	78	34	683	1.1	1.1	1020	2.1	1.6
**NCI**	264554	5190	72	42	2751	1.1	1.3	4987	2.0	1.8
**MLSMR**	343605	4450	77	28	330	1.0	0.9	1292	2.9	1.4
**KBD**	22615	688	89	33	64	1.9	1.3	221	4.3	1.8
										
		**SlogP**			**RB**			**nO**		
**WDI**	61368	1940	2.2	2.7	2244	5.9	4.3	1488	4.2	3.2
**NPD**	103668	1387	2.6	2.0	836	5.3	3.0	603	3.8	1.9
**NCI**	264554	4903	2.0	2.3	6340	4.6	3.2	4908	3.0	2.1
**MLSMR**	343605	6776	2.8	1.4	1100	6.1	2.6	2438	3.0	1.7
**KBD**	22615	487	3.4	1.8	465	6.1	3.3	683	2.4	1.9

Also included is the number of compounds (Total), outliers, average (Avg), and standard deviation (SD).

The statistical analysis of all descriptors shows that, with the exception of nO, the average values of all the other descriptors, i.e., (Wt, RB, HBA, HBD, TPSA, SlogP, Rings, and nN) were higher for kinase active compounds than for the compounds contained in the WDI, NPD, NCI, and MLSMR databases. Of note, the average RB value of the MLSMR compounds and average TPSA value of the WDI compounds were similar to that of the kinase active compounds. Kinase actives had the lowest average value for nO while compounds from the WDI database had the highest nO value. This analysis showed that, on average, the kinase active compounds were heavier (more weight), more flexible (more RB), more polar (more nN, HBA, and HBD) and more hydrophobic (more SlogP and rings) than the compounds in the other databases.

### Kinase-likeness

**Figure 2:**
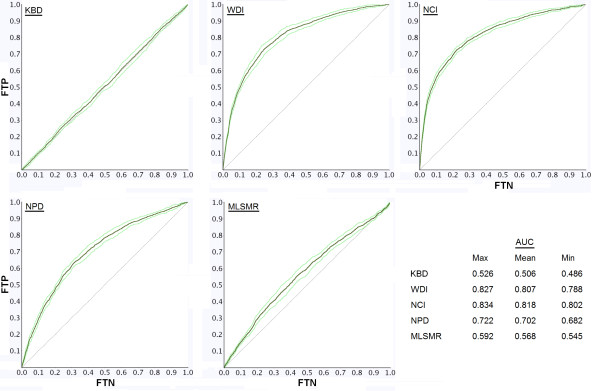
**ROC plots of five databases: KBD, WDI, NCI, NPD, and MLSMR**. The Y-axis indicates the fraction of true positives (FTP) and the X-axis indicates the fraction of true negatives (FTN). The table in the lower right shows the calculated maximum (upper light green line), mean (dark green line) and minimum (lower light green line) AUC values of the ROC plots based on the 10 different calculations for each database. AUC values closer to random (0.50) signify more kinase-likeness, meaning that kinase-likeness score is unable to distinguish the 1% of kinase active compounds that were mixed in from the other compounds in a given database. This indicates that those compounds have physicochemical properties that are similar to kinase active compounds. A diagonal reference line (area under the curve = 0.50) defines points where kinases are not discernible from the test database compounds. As validation, randomly selected kinase compounds were mixed in with the KBD (upper left plot) and an average AUC of 0.5 was observed.

We utilized the distinctive properties of kinase active compounds to develop a kinase-likeness scoring function. Based on the distribution of the descriptor values of kinase actives from the KBD, we calculated a kinase-likeness score, *KLS*, for each small molecule in the KBD, WDI, NPD, NCI, and MLSMR databases (Equation 1). This score reflects how similar each compound is with respect to the physicochemical properties of known kinase actives. To calculate how kinase-like these databases were, we generated an ROC plot (as described in the Methods section) based on the sorted *KLS*. This is shown in Figure 2. If a small random set of kinase actives is seeded into the KBD, and VS is performed using the *KLS *to calculate an ROC AUC, this should produce an AUC value near 0.5. Our results showed this to be 0.506 (upper left plot in Figure 2). This means that the *KLS *is unable to distinguish the 1% mixed kinase active compounds from other kinase actives in the KBD; in other words, the compounds in the KBD are kinase-like. This is an obvious result but provides a good way to test if databases other than the KBD contain a large number of kinase-like compounds. If similar screening experiments with databases other than the KBD result in computed AUC values near 0.5, these databases would be deemed kinase-like. This in turn, indicates that these databases are composed of compounds that primarily have physicochemical properties similar to that of kinase active compounds. Using these ROC plots and associated AUC values, we can determine which databases are kinase-like. Here we focused only on the NCI, NPD, and MLSMR databases because of their common usage in screening for kinase targets (Figure 2). The WDI database was used as a comparison based on the diversity of compounds within it.

Table 2 shows the p values computed using a two-sided Student's *t *test for comparison of AUC values between the KBD and the other databases (WDI, NPD, NCI, and MLSMR). Note that all p values were < 0.05, indicating that the AUC distributions for the NCI, WDI, NPD, and MLMSR databases were significantly different than the AUC distribution for the KBD.

**Table 2:** The p values computed from a two-sided Student's *t *test for comparison of AUC distributions between the KBD, and WDI, NCI, NPD, and MLSMR databases.

	WDI	NCI	NPD	MLSMR
**AUC**	0.807	0.818	0.702	0.568
**p value**	4.9E-05	4.3E-05	2.7E-04	2.6E-02

There is a statistically significant difference between the KBD AUC distribution and the AUC distributions of WDI, NCI, NPD, and MLSMR respectively because the p values for the comparisons are all < 0.05.

From our ROC analysis, based on AUC values, we divided these databases into high, medium, and low kinase-like categories. High kinase-likeness databases have AUC values near 0.5. This is the case for the MLSMR database with an AUC value of 0.57. Low kinase-likeness databases have AUC values closer to 1.0. This is the case for the NCI (AUC = 0.82) and the WDI (AUC = 0.81) databases. Medium kinase-likeness databases have AUC values near 0.75. This is the case for the NPD database (AUC = 0.70). The high AUC result for WDI is expected since it contains marketed and in-development small molecule drugs that bind to a wide variety of protein targets. We believe that the lower AUC value for the MLSMR and the comparatively higher AUC value for the NCI database is a consequence of the more recent assembly of the MLSMR database compared to the NCI database. The MLSMR initiative started in 2005, whereas the NCI has been operational since the late 1950's. Research on kinase inhibitors has only ramped up in recent decades and consequently MLSMR, being the newer of the two databases, contains more kinase active compounds than the NCI. The NPD sits right in the middle with an AUC of 0.7. The hypothesis is that although NPD represents a hugely diverse set of natural product compounds (due to the large size range of the compounds), some portions of NPD have been optimized for kinase-likeness. Therefore, the NPD AUC value is not quite as high as the AUC values of the WDI or MLSMR databases (because of the kinase-like portions), but it is not as low as the KBD either (because of the diversity).

Using *in silico *rules prior to screening can help focus efforts on databases appropriate to protein targets of interest. The results presented here suggest that, when screening for potential kinase actives, it would be most efficient to probe the databases in the following order: MLSMR > NPD > WDI ≈ NCI.

To support our AUC-based results for the ranking of VS databases, we further analyzed them using the *KLSC *score (Equation 2), which is based on the similarity between the correlation matrix of a test database and the KBD. The calculated correlation matrix of nine physicochemical descriptors is shown in Table 3 for KBD compounds.

**Table 3:** Correlation matrix between nine physicochemical descriptors for the KBD.

Property	RB	HBA	HBD	SlogP	TPSA	Rings	nN	nO
**Weight**	0.70	0.44	0.14	0.20	0.50	0.54	0.40	0.40
**RB**		0.39	0.13	0.01	0.52	0.00	0.29	0.43
**HBA**			0.45	-0.12	0.62	0.22	0.37	0.50
**HBD**				-0.11	0.51	0.08	0.21	0.23
**SlogP**					-0.44	0.29	-0.13	-0.26
**TPSA**						0.05	0.38	0.66
**Rings**							0.37	-0.04
**nN**								-0.29

The correlation matrices of the other databases (WDI, NPD, NCI, and MLSMR) are provided in the Additional File 2. Ohno et al. has recently applied a similar scoring function, called the Drug Like Score (DLS), to compare commercially available compound libraries, clinical candidate libraries, and several other chemical libraries with a launched drug library to calculate drug likeness based on six physicochemical descriptors [56].

The calculated *KLSC *values for the NCI, WDI, NPD, and MLSMR databases are 0.114, 0.161, 0.079, and 0.066, respectively. A lower *KLSC *value signifies higher kinase-likeness for a database. The *KLSC *values shown above reiterate what we found before, i.e., MLSMR contain the most kinase-like active compounds of all the databases studied. Overall, the ranking of kinase-likeness based on *KLSC *values is MLSMR > NPD > WDI ≈ NCI. This is similar to that observed in ROC-based AUC analysis described in the previous section and shown in Table 2. An additional statistical test based on Kolmogorov-Smirnov methodology (KS test) [71] is shown in Additional File 3 along with the distribution of KLS values for all the studied databases. The *k *value obtained by KS test further confirmed the ranking of databases obtained by both the AUC based ranking and the *KLSC *value based ranking.

### Application in Docking-based Virtual Screening

With the aim of demonstrating the practicalities of the kinase-likeness scoring function, two datasets were created: a kinase-focused set and a random set. Both these datasets contain 10,000 small molecules (details in Methods section). To test which of these two datasets was more enriched in kinase-binding compounds, we performed VS of each against three different kinase targets selected from three different branches (families) of the human kinome tree. The criteria for selecting these kinase drug targets was that, 1) they should have been used in previous structure-based VS studies, 2) they should have been crystallized with reasonable resolution (≤ 3.0Å) with bound ligand, and 3) each should be from a different kinase family. With these criteria, we selected B-Raf to represent the tyrosine kinase-like (TKL) family, mitogen-activated protein kinase-activated protein kinase 2 (MK2) to represent the Ca^2+^/calmodulin-dependent protein kinases (CaMK) family, and cyclin-dependent kinase 2 (CDK2) to represent the CMGC kinase family. The PDB codes for these proteins are 2FB8[72], 2PZY[73], and 1OIT[74] respectively.

**Figure 3:**
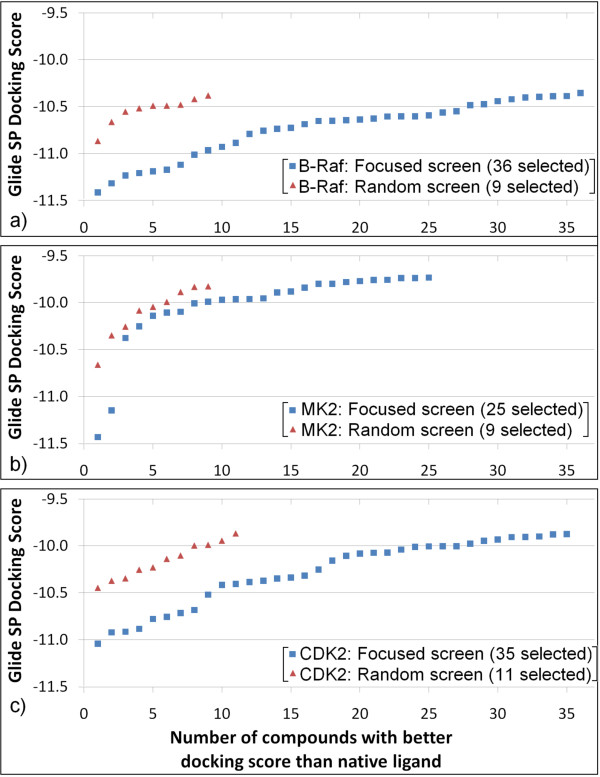
**Top virtual screening hits from the focused set database (blue squares) and the random set database (red triangles) for three selected kinase targets: a) B-Raf; b) MK2; and c) CDK2**. For each target, only those selected docking hits are shown that yielded a better docking score than the native ligand docking score for both screening libraries, i.e., the focused set and the random set. A significant difference was observed in the number of selected hits between these library sets, with better enrichment of the focused set of compounds.

A docking-based VS, as explained in the Methods section, was performed using both the kinase-focused compound set and the random compound set. The outcomes were analyzed in terms of number of "hit" compounds, defined as compounds that scored better than the docked native ligand in each respective crystal structure of the selected targets. The results are shown in Figure 3. In each case, three to four times more hits were obtained from the focused compound set than from the random compound set. Of note, many of the top hit compounds from the focused set yielded a much better docking score than the top hits from the random set. These results demonstrate that the compounds from VS of the focused set are not only more enriched for kinase-like compounds than the random screen, but also interact better with the kinase binding pockets.

To show that physicochemical property-based focused libraries are more diverse than structure-based focused libraries, we created three focused libraries based on the kinase drug Sunitinib: scaffold-based, similarity-based, and physicochemical properties-based. All three focused libraries were extracted from the CNC-AMS database of ~ 7 million purchasable compounds. In the first case, only the cyclic scaffold of drug Sunitinib was taken into account when performing scaffold screening of CNC-AMS. A total of 44 compounds were found to contain similar cyclic scaffold. In the second case, the full structure of Sunitinib was used for screening and the top 100 similar compounds, based on Tanimoto similarity value, were selected. In the third case, the KLS scoring function was applied on all the compounds of CNC-AMS and top 100 scoring compounds were selected. The average distance (1-similarity) between each pair of molecules was 0.69, 0.74, and 0.85, for scaffold-base, similarity-based, and the physicochemical property-based (KLS score) focused libraries, respectively. For comparison a collection of 100 randomly select compounds from CNC-AMS shows average distance of 0.88. This proves that the physicochemical property-based focused library is more diverse than structure-based focused libraries. In fact, the average distance value of 0.85 for the physicochemical property-based focused library generated from CNC-AMS is as diverse as a collection of random selected compounds from CNC-AMS.

## Conclusion

Kinases represent one of the most important and therapeutically relevant drug target families involved in diseases like cancer and various other neurodegenerative, infectious and inflammatory diseases. In the past few years, various computational methodologies have been employed to facilitate the kinase drug discovery effort through the screening of small compound databases. Many of these methodologies focus on steps that reduce the size of screening databases. For example, the most common types of pre-screening filters are based on selective and preferential kinase scaffold-based filtering and 2D- and 3D-based similarity filtering using the templates of known kinase active compounds. In the work presented here, we demonstrated another easily calculable and amenable statistical methodology for the generation of virtual kinase-focused libraries.

This methodology is based on the observation that various physicochemical properties of known kinase active compounds such as molecular weight, polar surface area, and rotatable bonds are unique for this structural target class and can be used to differentiate kinase active compounds from molecules of other structural classes. This method calculates a kinase-likeness score based on property distribution ranges of nine physicochemical descriptors derived from known kinase active compounds. These physicochemical properties are molecular weight, SlogP, TPSA, rotatable bonds, hydrogen bond acceptors and donors, number of rings, number of nitrogen and oxygen atoms. Previous studies of making kinase-focused libraries use scaffold-based and similarity-based filters, which are primarily inclusive filters. These methodologies obtain enrichment by including molecules containing scaffolds identified in known actives. Such methods are popular, but the collected compounds lack structural diversity, since they are all based on either the same or similar known scaffolds. By comparison, our physicochemical filtering scheme achieves enrichment by including molecules that exhibit certain desirable physicochemical properties. Since this method relies solely on the physicochemical properties, any selection done using it becomes independent of known scaffolds. Hence the possibility of finding novel scaffolds that are structurally unrelated to known kinase actives is greatly increased. Using an example of kinase drug Sunitinib, we showed that focused libraries based on physicochemical property screening are more diverse than those generated by structure based techniques.

Our method can be used to prioritize small molecule databases for kinase-based virtual screening. Subsequent to database prioritization, the method can be used to constrain the size of screening databases and create focused libraries. The method can even be used to guide the *de novo *design of molecules prior to synthesis and testing. Extension of this method to improve the quality of other focused libraries is easily possible; the scoring function is readily modifiable since new physicochemical parameters may be added and less useful ones may be deleted.

Using our kinase-likeness score and a validated correlation matrix-based score for comparison, we showed that the MLSMR database compounds are much closer to kinase actives, in terms of physicochemical properties, than compounds in the NCI and NPD databases. We also developed and tested a kinase-focused library based on our kinase-likeness score and showed its superior enrichment for three specific kinase targets from different branches of the human kinome, relative to random screening of a collection of compounds. In each case, the focused library showed three to four times more enrichment compared to random screening of compounds. Given the demonstrated performance of our kinase-like scoring function for generating enriched libraries, we suggest that it may be useful as a first step in kinase-targeted virtual screening workflows.

## Competing interests

The authors declare that they have no competing interests.

## Authors' contributions

NS implemented the methods and conducted the experiments with assistance from HS, SC, MDMA, AW and GT. All five participated in study's design, coordination and manuscript drafting, and gave the final approval of the version to be submitted.

## Supplementary Material

Additional file 1 **Distribution maps of physicochemical descriptors**. Statistical parameters based on the distribution maps of nine calculated physicochemical descriptors for five databases (DB): WDI, NPD, NCI, MLSMR, and KBD. Also included is the number of compounds (Total), outliers, average (Avg), standard deviation (SD), and P10-P90 (range covering 80% of the compounds) values. Click here for file

Additional file 2 **Correlation matrix of physicochemical descriptors**. Correlation matrix between nine physicochemical descriptors for the WDI, NPD, NCI, and MLSMR databases. Click here for file

Additional file 3 **Distribution map of Kinase-likeness score (KLS)**. Distribution of KLS for the WDI, NPD, NCI, and MLSMR databases. Click here for file
